# Prescribing practice for malaria following introduction of artemether-lumefantrine in an urban area with declining endemicity in West Africa

**DOI:** 10.1186/1475-2875-9-180

**Published:** 2010-06-24

**Authors:** Joseph U Okebe, Brigitte Walther, Kawsu Bojang, Silaba Drammeh, David Schellenberg, David J Conway, Michael Walther

**Affiliations:** 1 Malaria Programme, Medical Research Council (UK), Atlantic Boulevard, Fajara, PO Box 273 Banjul, The Gambia; 2 Statistics Department, Medical Research Council (UK), Atlantic Boulevard, Fajara, P O Box 273 Banjul, The Gambia; 3 Divisional Health Team, Western Division, Department of State for Health, Kanifing Municipal Council, The Gambia; 4 Disease Control and Vector Biology Unit, Department of Infectious and Tropical Diseases, London School of Hygiene and Tropical Medicine, Keppel Street, London WC1E 7HT, UK; 5 Pathogen Molecular Biology Unit, Department of Infectious and Tropical Diseases, London School of Hygiene and Tropical Medicine, Keppel Street, London WC1E 7HT, UK

## Abstract

**Background:**

The decline in malaria coinciding with the introduction of newer, costly anti-malarials has prompted studies into the overtreatment for malaria mostly in East Africa. The study presented here describes prescribing practices for malaria at health facilities in a West African country.

**Methods:**

Cross-sectional surveys were carried out in two urban Gambian primary health facilities (PHFs) during and outside the malaria transmission season. Facilities were comparable in terms of the staffing compliment and capability to perform slide microscopy. Patients treated for malaria were enrolled after consultations and blood smears collected and read at a reference laboratory. Slide reading results from the PHFs were compared to the reference readings and the proportion of cases treated but with a negative test result at the reference laboratory was determined.

**Results:**

Slide requests were made for 33.2% (173) of those enrolled, being more frequent in children (0-15 yrs) than adults during the wet season (p = 0.003). In the same period, requests were commoner in under-fives compared to older children (p = 0.022); however, a positive test result was 4.4 times more likely in the latter group (p = 0.010). Parasitaemia was confirmed for only 4.7% (10/215) and 12.5% (37/297) of patients in the dry and wet seasons, respectively. The negative predictive value of a PHF slide remained above 97% in both seasons.

**Conclusions:**

The study provides evidence for considerable overtreatment for malaria in a West African setting comparable to reports from areas with similar low malaria transmission in East Africa. The data suggest that laboratory facilities may be under-used, and that adherence to negative PHF slide results could significantly reduce the degree of overtreatment. The "peak prevalence" in 5-15 year olds may reflect successful implementation of malaria control interventions in under-fives, but point out the need to extend such interventions to older children.

## Background

In malaria endemic areas, the lack of specific features and adequate laboratory diagnostic facilities often limits the capacity of health workers to establish a definitive diagnosis of malaria. Given the potentially lethal consequences of a missed diagnosis of malaria, case management guidelines had adopted a presumptive approach to treatment for the febrile child in areas considered endemic for disease [1]. Given the high disease burden, and the availability of cheap and effective anti-malarials, this was considered a cost-effective approach to care [2] and is acknowledged to have contributed to the reduction in morbidity and mortality in preschool children [3,4].

With reports of declining incidence of malaria [5-10] and now almost universal introduction of artemisinin-based combination therapy (ACT), there have been increasing calls for improving the way diagnosis and case management for malaria is conducted, with a flurry of diagnostic options [11-14]. This follows because it has been well documented that presumptive anti-malarial treatment of febrile cases results in considerable overdiagnosis and treatment across varying transmission settings [15-21]. For instance, a large scale study in north-east Tanzania revealed that 54% of hospital admitted patients treated for malaria had no evidence of *Plasmodium *infection as assessed by expert slide microscopy [19]. Since these patients were usually not investigated or treated for other potentially life-threatening diagnoses, the case fatality rate in the malaria parasite negative group presumptively diagnosed was 1.75-fold higher than in the parasite positive group.

With declining incidence of malaria, adherence to the reflex to treat any febrile case for malaria is likely to put proportionately more patients at risk of not receiving appropriate treatment. Also, the indiscriminate use of ACT has major cost implications and potentially increases the risk of an emergence of resistance [22,23]. To address these issues, accurate data are needed on the degree of overtreatment from presumptive diagnosis in different populations [24,25].

The bulk of the literature on the subject reports from studies in East Africa in areas with different degrees of malaria transmission. The aim of this study is to provide comparative evidence from a West African country with a distinctly seasonal malaria transmission pattern. The study, therefore, investigated prescription practices immediately following artemether-lumefantrine (AL) introduction as first-line treatment in the Gambia, during and outside of the annual malaria transmission season. The implications of these findings on case management and diagnostic options are discussed.

## Methods

### Health facility survey

Two cross-sectional surveys were carried out at two primary health facilities (PHFs) serving predominantly urban populations within the Western Division of The Gambia, to coincide with the periods of low transmission (May to July) and peak malaria transmission (August - October) respectively. The study was carried out in 2008 shortly after the introduction of AL into the health system as the first-line treatment for uncomplicated malaria. Both PHFs have laboratory facilities for blood slide microscopy and the outpatient clinics are run by state enrolled nurses trained in the use of the national malaria treatment guideline. The guideline reflects the WHO guideline in identifying symptoms which could indicate malaria and encourages the use of microscopy for diagnosis where available but permits presumptive treatment on the basis of clinical suspicion without a confirmatory test since not all health facilities in the country have supporting laboratories [26]. Eligibility for this study was based on a patient seen and treated for malaria by the attending health worker; no attempt was made to interfere with the consultation process. Patients were sequentially enrolled however; those already on anti-malarials prior to presentation and those, requiring in-patient care were excluded.

Written informed consent was obtained after consultation, and information on presenting complaints, duration of illness, diagnosis made and treatment given were collected onto case report forms. In addition, a finger prick blood sample was collected from each participant for a blood slide and this was read at the Medical Research Council's haematology (reference) laboratory at Fajara. A film was declared negative if no asexual form of *P. falciparum *was seen on examining 100 high-power fields.

The outpatient register used to record attendance and treatments was reviewed to document the total number of people seen and treated for malaria at the PHFs throughout the study period since study recruitment was restricted to working hours on week days only.

### Sample size determination

Studies elsewhere evaluating the proportion of slide negative cases who received anti-malarial treatment have reported values between 62% [15] in areas of perennial transmission and 86% [27] for areas with unstable low transmission. The study hypothesized that prescription patterns would differ during outside the transmission season and aimed to detect at least a 15% difference between seasons. For a 5% significance level with 80% power, and taking into account incomplete data entry, a minimum estimate of 207 patients were enrolled at each time point; 414 in all. This sample size took into account possible differences in the age distribution of patients between seasons and balanced selection of patients into predefined age strata at the point of recruitment, based on the age distribution of malaria cases documented in the outpatient registers for the previous year.

### Data processing and analysis

Microsoft Access 2003 (Microsoft Inc, Redmond, WA, USA) was used for double entry, validation and verification of the data. The analysis was carried out using STATA, version 10 (Stata Corp, College Station, Tx, USA). The outcome for the study was the proportion of cases that received anti-malarial treatment, but had a negative blood slide result from the reference laboratory reading. Logistic regression models including age groups (<5, 5-15, >15 years), season and interaction terms for age groups and season were performed. Subgroup analysis was carried out for children (<5 and 5-15 years). Sensitivity and specificity of the health centre slide readings compared to the MRC readings was evaluated in the subgroup of patients who had a request for microscopy made at the health facility.

### Ethical approval

The study was approved by the Gambian Government/MRC Joint Ethics Committee.

## Results

### Description of sample population

**Figure 1:**
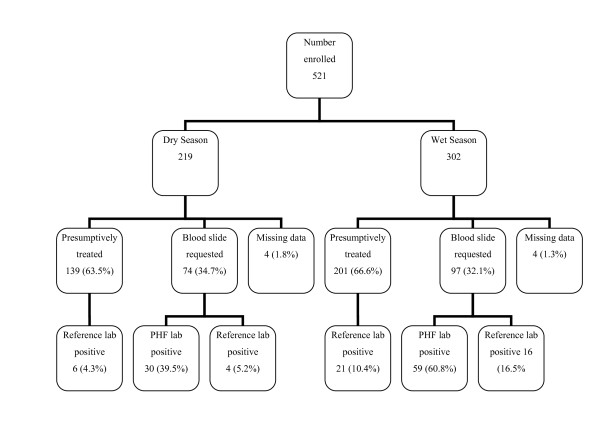
**Distribution of patients between seasons showing numbers (%) in the different comparison groups studied**.

About 8,410 patients were recorded as being treated for malaria at both facilities during the study period; 1,502 in the dry and 6,908 in the wet seasons respectively. A total of 521 patients treated for malaria were enrolled, but analysis was carried out using data for 513 patients; 215 and 298 during the dry and wet seasons respectively (eight entries were excluded due to incomplete data)(Figure 1). The distribution of patients by age category and sex in both survey periods is shown in Table 1. All but five patients reported a history of fever with a median duration of three days (range 1 - 60 days) before presentation, although temperatures were not measured routinely at the PHFs.

**Table 1:** Age and sex distribution of patients by season

	Season N (%)	
Age category (years)	Dry	Wet
<5	89 (41.4)	124 (41.6)
5-15	54 (25.1)	78 (26.2)
>15	72 (33.5)	96 (32.2)
Sex		
Male	103 (47.9)	136 (45.6)
Female	112 (52.1)	162 (54.4)

### Health facility performance: slide requests and results

A request for microscopy was made in 33.2% (173) of those enrolled (Table 2). Slide requests were significantly more common for children (0-15 yrs) than adults (>15 years) in the wet season (p = 0.003), but not during the dry season (p = 0.64). Among the adults, a higher proportion received slide requests during the dry season compared to the wet season (p = 0.018). While the proportion of slide requests was similar for children below and above five years of age in the dry season (p = 0.205), a significantly higher proportion of those under five years received a request compared to those above five years in the wet season (p = 0.022).

**Table 2:** Health centre slide requests

Age category	Dry season [n/N (%)] †	Wet season [n/N (%)] †	OR (95% CI)	p-value
<5 years (213)	27/89 (30.3)	55/124 (44.4)	1.83 (1.03-3.25)	0.039
5 - 15 years (132)	22/54 (40.7)	22/78 (28.2)	0.57 (0.27-1.19)	0.135
>15 years (168)	27/72 (37.5)	20/96 (20.8)	0.44 (0.22-0.87)	0.018

†N-total number per age category/season

* Requests in the wet season compared to the dry season

Overall, a blood slide was scored positive in 52.4% of the children and 48.9% of adults tested in both seasons (Table 3). Considering that all those enrolled received anti-malarials despite their test result, this would infer an overtreatment rate of 48.6% based on the PHF laboratory result. Ninety-five percent of those treated received the recommended AL. There was no significant association between test result and anti-malarial prescribed (p = 0.060).

**Table 3:** Proportions and odds of slide positive results at the PHF laboratories

Season	Age category	Sample	Slide positive (%)	OR (95% CI)	p-value
Dry	<5 years	27	15 (55.6)	1	
	5 - 15 years	22	10 (45.5)	0.67 (0.22 - 2.07)	0.483
	>15 years	27	5 (18.5)	0.18 (0.05 - 0.62)	0.007
Wet	<5 years	55	24 (43.6)	1	
	5 - 15 years	22	17 (77.3)	4.39 (1.42 - 13.60)	0.010
	>15 years	20	18 (90)	11.63 (2.46 - 55.05)	0.002

Notable differences were observed in the proportion of confirmed cases in the different age categories, and in both seasons. During the dry season the proportion of positive cases declined with age, while in the wet (transmission) season, the highest proportion of positive cases were reported in the age group 5-15 years, with children in that age category being 4.4 times more likely to have a positive result compared to those <5 years old. Also, children within the 5-15 years age group had significantly more positive cases in the wet season compared to the dry season (p = 0.03).

### Evaluating malaria treatment based on reference microscopy results

**Figure 2:**
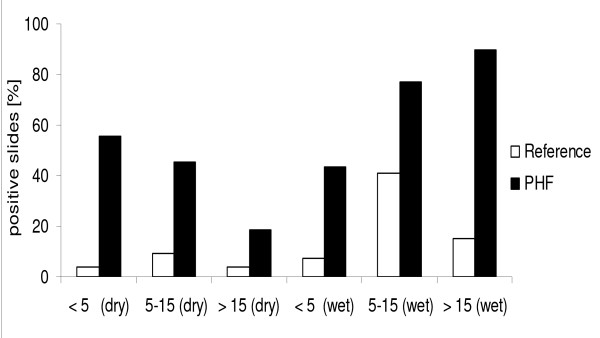
**Comparison of slide reading results between laboratories for both seasons, stratified by age category**.

The reference slide readings suggest considerably lower parasite prevalence amongst those treated for malaria. Only 9.2% of the 512 patients enrolled and treated for malaria had parasitaemia at the time of diagnosis; 4.7% (10/215) in the dry season and 12.5% (37/297) in the wet season (p = 0.003, Table 4). Conversely, this infers that 95.3% (95% CI: 92.5% - 98.2%) and 87.5% (95% CI: 83.8% - 91.3%) of those who received anti-malarials in the dry and wet seasons respectively may have been treated wrongly as judged by the reference slide reading. This averages to an overtreatment rate of 90.8% (95% CI: 90.2% - 91.4%) for both seasons. The reference slide reading confirms that the odds of a positive slide result are highest in the 5-15 years age category across both seasons. Figure 2 compares the slide reading results from both laboratories stratified according to age groups according to season.

**Table 4:** Reference laboratory results for malaria parasitaemia in both seasons

MRC slide results	N = 512 (%)*	ORs	95% CI	p-value
DRY	10/215 (4.65)			
<5	1/89 (1.1)	1		
5-15	7/54 (13.0)	13.11	1.565 - 109.73	0.018
>15	2/72 (2.8)	2.51	0.223 - 28.30	0.455
WET	37/297 (12.46)			
<5	9/123 (7.32)	1		
5-15	17/78 (21.8)	3.53	1.485 - 8.390	0.004
>15	11/96 (11.5)	1.64	0.650 - 4.132	0.295
Total	47/512 (9.2)			

* One slide washed off during preparation

The proportion of patients overtreated according to age is shown in Table 5 and indicate that children <5 years are most significantly misdiagnosed and treated for malaria compared to other age groups for both seasons. Extrapolation of the numbers of cases unnecessarily treated for malaria based on the number of malaria diagnoses documented at the recruitment site over the study period would suggest that 7,636 (95% CI: 7,586 - 7,686) of the 8,410 may have received anti-malarials for illnesses other than malaria.

**Table 5:** Overtreatment estimates in both seasons across age groups in the sample

	Age category	% overtreatment (95% CI)
Dry season	<5	98.9% (96.6 - 101.1)
	5-15	87.0% (77.8 - 96.4)
	>15	97.2% (93.3 - 101.1)
Wet season	<5	92.7% (82.1 - 97.4)
	5-15	78.2% (68.8 - 87.6)
	>15	88.5% (82.1 - 90.3)

### Accuracy of health facility slide reporting

To determine the accuracy of the PHF slide results, they were compared to those obtained at the reference laboratory used as the standard.

During the wet season, the sensitivity of slide reporting was quite high at 93.8% but the ability to identify true non-malaria cases was low with a specificity of 45.7%. Though sensitivities did not differ significantly between seasons (p = 0.37), specificity was significantly higher during the dry season (p = 0.037, Table 6). While the positive predictive value of a PHF slide was low, being 10% during the dry season and 25.4% in the wet season, the negative predictive value (NPV) remained high being 97.8% and 97.3% in both the dry and wet season, respectively.

**Table 6:** Sensitivity and specificity of health facility results by season

	Sensitivity (95% CI)	Specificity (95% CI)
Dry season	0.750 (0.326- 1.00)	0.625 (0.513 - 0.737)
Wet season	0.937 (0.819- 1.00)	0.457 (0.348 - 0.565)
Comparison between seasons	p = 0.37	p = 0.037

## Discussion

Presumptive diagnosis of malaria previously advocated for malaria endemic areas may have always resulted in some degree of overtreatment. With the decline in malaria prevalence reported from several parts of sub-Saharan Africa [9,10,28,29], adherence to this approach is likely to increase the degree of overtreatment and inevitably a failure to treat alternative causes of fever. Indeed, studies in critically ill patients treated for "malaria" showed significantly higher mortality in those with a negative slide compared to those with a positive slide [19,30]. An economic layer is added to this problem with an increasing number of countries now using relatively costly ACT currently as the first line of defence against malaria. These are important reasons to develop treatment strategies targeting those who actually have malaria and requires a better understanding of the phenomenon of malaria overdiagnosis.

Overtreatment has been reported from many parts of East Africa, mainly from areas with low to moderate or high perennial transmission [15,16,19,21]. The pattern is likely to vary across different epidemiologic settings, but little has been known from areas where malaria incidence has dropped to very low levels [31], and data are lacking from West Africa. Here, data on prescription practices in the Gambia, where malaria is highly seasonal [32] and where incidence has declined to unprecedentedly low levels [9] are presented.

The observations indicate that PHF workers under-utilize the available laboratory facility in making decisions on diagnosis. Despite the availability of slide reading at the health centres, a slide was only requested in about 1/3 of the cases diagnosed with malaria. Interestingly, this proportion remained stable throughout the year despite the fact that substantially more patients are seen during the wet than in the dry season and suggests that the capacity to perform slide reading is unlikely to be the limiting factor.

The low use of the laboratory facilities supports other reports [24,33], and may in part be explained by the mistrust in the quality of the slide reading [34], which may be justified. While the sensitivity of the microscopic diagnosis performed at the PHF was acceptable compared to the reference slide, the specificity was unacceptably poor, resulting in a high number of "false positives". Thus, similar to reports from East Africa [35], slide reading at the PHF has an extremely poor positive predictive value, which argues in favour of presumptive treatment.

However, as has been shown previously [19,31], a negative slide did not necessarily exclude a patient from receiving malaria treatment. The negative predictive value was high in both the dry season (97.8%) and the wet season (97.3%), which would have allowed exclusion of slide negative cases from anti-malarial treatment with high confidence. Adherence to the policy to withhold malaria treatment from patients with a negative slide result could, therefore, save a significant amount of overtreatment without significantly increasing the risk of missing true malaria cases but achieving compliance with this policy is obviously challenging.

The data presented here indicate that the health workers tend to target children less than five years of age for slide requests, particularly during the transmission season. While the focus on this age group probably reflects the impact of programmes such as the Integrated Management of Childhood Illnesses (IMCI), the increased use of parasitological diagnostics in this age group (for which the IMCI guideline advocated presumptive treatment), may be influenced by more recent local guidelines that promote the use of diagnostics where available. It is conceivable that the recommendation to perform parasitological diagnostics is perceived primarily as an additional layer of measures aimed to improve care in those already identified as risk groups.

However, microscopic diagnosis of blood slides from all participants at the reference laboratory show that, among those for whom a slide reading was requested, the highest prevalence of malaria parasites was in 5-15 year old children; these being 3.5 and 13.1 fold more likely to have parasitaemia in the wet and dry seasons respectively than children under the age of five years. To some extent this may be confounded by the fact that care-givers are more likely to bring younger children to the PHF when they are febrile, and that febrile disease due to a variety of causes other than malaria may simply be more prevalent in younger compared to older children. However, a cross sectional survey carried out recently in healthy Gambians similarly identified the peak parasite prevalence occurring in 10-15 year olds [9]. It would be tempting to speculate that this may be the result of consequent implementation of malaria control strategies in under fiver year olds, but up to date information on bed net coverage in the study area is lacking. Taken together, this suggests that in areas of low malaria transmission the target group for malaria control measures should be enlarged to include children up to the age of 15 years.

The most remarkable observation, however, is that in the wet season, 87.5% had no detectable parasitaemia on the reference slide. During the dry season 4.6 times less patients received malaria treatment, but among those, the proportion of unnecessarily administered anti-malarials was even higher, being 95.3%. Several reports from East Africa have recently documented malaria overdiagnosis, and - although only few studies simultaneously measured transmission by means of entomological inoculation rates (EIR) - it appears that overdiagnosis seems to increase as transmission declines. In areas of perennial [15] and moderate [19] transmission in Tanzania, 62% and 54% of malaria treatments were found to be unnecessary, respectively, whereas overtreatment rates of 86% [27] and 75% [16] were reported from areas in East Africa where transmission was classified as low and unstable. One recent study in Tanzania measured both transmission and the degree of overtreatment at the same time and determined that where the EIR was 0.69 by mosquito sampling, 99.6% of patients receiving anti-malarials had no slide-detectable parasitaemia [31]. Comparable data here for the Gambia [9,36] for the year preceding this survey, supports the notion that over-prescription may relate inversely with a decline in the level of malaria transmission.

In order to treat patients appropriately and to ensure the sustainability of ACT and to protect the "useful lifespan" of such drug combinations, the new guidelines from WHO recommend that a laboratory test should be performed before treating [37]. Therefore, efforts are being made to improve access to parasitological diagnostics. Presently, the options available are to increase capacity to carry out microscopy or the widespread use of rapid malaria diagnostic test kits (RDTs).

Attempts to step up the capacity of slide reading are likely to be of limited success, as microscopy depends on reliable electricity supply and requires considerable skill and expertise. RDTs in a dipstick format appear to be an attractive alternative for parasitological diagnosis. RDTs do not require particular skills or electricity and can be performed at the point of care by the PHF worker. However, procurement of RDTs is probably only a modest proportion of the cost of integrating RDTs into a health system. The challenges of forecasting RDT needs, managing procurement and distribution through the supply-chain should not be underestimated.

Discussions about the best way forward have stimulated a vibrant debate with strong advocacy in favour [38] and against [39] the rapid implementation of RDT. While RDTs perform at least as well as microscopy in diagnosing malaria, it appears clinicians are reluctant to refrain from treating for malaria even after a negative test [40]. Surprisingly, the fact that RDTs can be performed by the PHF worker did not enhance adherence to the test result, and in particular children under five years with a negative RDT were more likely to be prescribed an anti-malarial than those with a negative slide [40,41]. Mathematical modelling using data across a broad range of malaria transmission intensities concludes that RDTs can be cost-effective compared to presumptive treatment, but these calculations assume that prescribers treat according to test results [42], and there are conflicting data as to what extent this could be the case in real life. In Burkina Faso, a similar amount of overtreatment was found in patients managed clinically or with RDT, indicating that test results were simply ignored [43]. In a Kenyan trial, prescribing of ACT largely followed the results however despite a negative test, 75% of children under five years and 61% of those over five years still received an anti-malarial [44]. In an area of Zanzibar with parasite prevalence ranging from 10% to 50%, the use of RDTs improved treatment and health outcomes, without increasing the costs for patient management [45].

It is important to note that revised WHO guidelines are not explicit whether or not to withhold anti-malarials from patients where there is a clinical suspicion of malaria but a negative test result. Here, the health care worker's decision depends on his/her trust into the quality of the test and his/her fear of the implications of a missed diagnosis. These situations often result in the half-hearted prescription of an anti-malarial other than the first-line drug [44].

Thus, whether RDTs have the potential to become a cost-effective means to improve the management of febrile cases will depend on successful strategies to promote adherence to the result, and has to overcome the labelling and treatment of all febrile illnesses as "malaria". PHF workers need to be toned to make efforts to consider other, often more difficult to establish, diagnoses and to convince caregivers where a fever has no serious cause.

## Conclusions

Despite their availability, a consistent pattern of underuse of diagnostic facilities was seen. The proportion of patients likely to receive an anti-malarial for a slide negative result was very high in both seasons. In the context of other published reports this suggests the tendency to overprescribe has increased with recent declines in transmission. While RDT based diagnostic approaches have the potential to be cost-effective, adherence to test results remains its major determinant. Behaviour-change communication campaigns will be needed to encourage withholding of malaria treatment from patients with a negative diagnostic test, thus saving costs without increasing the risk of missing out on the true malaria cases.

## Competing interests

The authors declare that they have no competing interests.

## Authors' contributions

JUO conceived and designed the project, lead the field work, analysed the data and drafted the manuscript; KB and SD helped with the field work and were involved in the review of the manuscript; BW helped with the statistical analysis of the data; DS and DJC provided input into the study design and drafting of the manuscript; MW conceived and designed the project together with JUO, helped with the analysis and drafted the manuscript. All authors read and approved the final manuscript.
